# Human Immunodeficiency Virus Type-1 (HIV-1) Transcriptional Regulation, Latency and Therapy in the Central Nervous System

**DOI:** 10.3390/vaccines9111272

**Published:** 2021-11-03

**Authors:** Joseph Hokello, Adhikarimayum Lakhikumar Sharma, Priya Tyagi, Alok Bhushan, Mudit Tyagi

**Affiliations:** 1 Department of Biology, Faculty of Science and Education, Busitema University, Tororo P.O. Box 236, Uganda; joseph.hokello@sci.busitema.ac.ug; 2 Center for Translational Medicine, Thomas Jefferson University, 1020 Locust Street, Philadelphia, PA 19107, USA; LakhikumarSharma.Adhikarimayum@jefferson.edu; 3 Cherry Hill East High School, 1750 Kresson Rd, Cherry Hill, NJ 08003, USA; priya.tyagi@gmail.com; 4 Department of Pharmaceutical Sciences, Jefferson College of Pharmacy, Thomas Jefferson University, Philadelphia, PA 19107, USA; Alok.Bhushan@jefferson.edu

**Keywords:** HIV, transcriptional regulation, latency, therapy, central nervous system

## Abstract

The central nervous system (CNS) is highly compartmentalized and serves as a specific site of human immunodeficiency virus (HIV) infection. Therefore, an understanding of the cellular populations that are infected by HIV or that harbor latent HIV proviruses is imperative in the attempts to address cure strategies, taking into account that HIV infection and latency in the CNS may differ considerably from those in the periphery. HIV replication in the CNS is reported to persist despite prolonged combination antiretroviral therapy due to the inability of the current antiretroviral drugs to penetrate and cross the blood–brain barrier. Consequently, as a result of sustained HIV replication in the CNS even in the face of combination antiretroviral therapy, there is a high incidence of HIV-associated neurocognitive disorders (HAND). This article, therefore, provides a comprehensive review of HIV transcriptional regulation, latency, and therapy in the CNS.

## 1. Introduction

Human immunodeficiency virus type-1 (HIV-1) remains one of the most serious public health challenges [1,2,3,4]. HIV-1 invades the brain soon following systemic infection. Several mechanisms have been suggested for HIV-1 entry into the central nervous system (CNS). However, the “Trojan horse hypothesis”, which states that HIV-1 infection of the brain occurs through migration of the infected cells across the blood–brain barrier (BBB), is the most favored. Although CD4+ T cells, as well as monocytes and macrophages, are the primary cellular targets for productive HIV-1 infection [5,6,7], in the brain, macrophages and microglia are the main cell types productively infected by HIV-1, and virus production in the CNS is not seen before the onset of acquired immunodeficiency syndrome (AIDS) [8,9]. Studies have also indicated that astrocytes, which are the most abundant and long-lived cell types, can also be infected by HIV-1 [10,11]. Although the HIV-1 infection of neurons and oligodendrocytes remains controversial [8], HIV infection is reported to result in the damage of neocortical neurons [12]. HIV-associated injuries of the CNS are believed to be mediated by numerous soluble factors that are released by microglial cells following HIV-1 infection. These soluble factors include both viral and cellular factors, some of which can directly induce neuronal injury and damage through interactions with receptors on neuronal membranes. However, the CNS responses to HIV-1 infection involve mechanisms that enhance the survival of neurons. In this review, we discuss the HIV-1 transcriptional regulation, latency, and the effects of highly active antiretroviral therapy in the CNS.

## 2. HIV Transcriptional Regulation in the CNS

The HIV-1 long terminal repeat (LTR) is responsible for HIV transcriptional initiation, and it contains *cis*-regulatory elements that specifically bind the transcription factors involved in the regulation of HIV transcription. The LTR is comprised of three distinct regions, including the unique 3′ (U3) end, repeated (R) sequence, and the unique 5′ (U5) end. The U3 is made up of elements that mediate RNA polymerase II (RNAP II) binding to the viral template DNA and the TATA box located at the −28 nucleotide position relative to the transcription start site. Two nuclear factor kappa beta (NF-κB) and three specificity protein 1 (Sp1)-binding sites are found at the 5′ end of the TATA box [4,13,14]. The HIV transcription is initiated from the LTR following the binding of a highly conserved 38-KD TATA box binding protein (TBP) to the TATA box sequence. TBP binding mediates the recruitment of additional transcription factors forming the preinitiation complex referred to as the TBP-associated factor (TAF). The TBP and TAF form a multiprotein complex referred to as transcription factor II D (TFIID), which is the minimal protein complex capable of inducing basal transcription from the HIV LTR.

**Figure 1:**
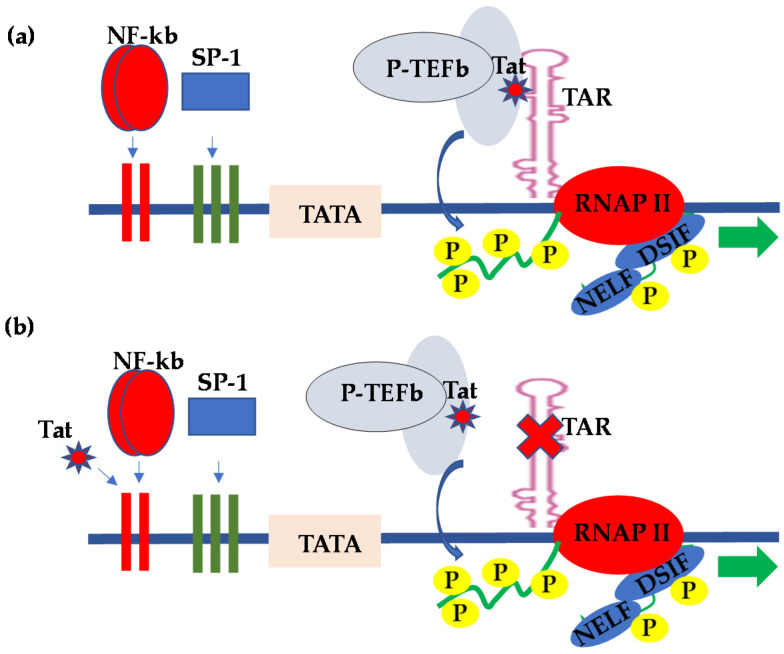
Molecular model for the (**a**) TAR-dependent and (**b**) TAR-independent regulation of HIV transcription.

It is, however, worth noting that the efficient initiation of transcription from the HIV LTR requires the interaction of TFIID with the upstream enhancer-binding transcription factors, such as NF-κB or the nuclear factor of activated T cells (NFAT) [14,15]. Transcription factor II H (TFIIH) then phosphorylates the C-terminal domain (CTD) of RNAP II within the transcription initiation complex in order to enhance HIV transcriptional elongation [14,16,17]. However, when the viral transcription trans-activator protein Tat is absent, HIV transcription is less efficient, because RNAP II stalls just a few nucleotides from the transcription start site following the promoter clearance. In the absence of the viral Tat protein, transcription initiation from the HIV LTR is efficient; however, the transcription is impaired, because the promoter engages poorly processive RNAP II, which disengages prematurely from the DNA template [18]. On the other hand, when Tat is present, the synthesis of viral messenger RNA (mRNA) is enhanced because of an increased elongation efficiency mediated by Tat [18,19,20,21]. In what is referred to as TAR-dependent HIV transcription, Tat acts by binding to an RNA element known as the transcription response (TAR) element, which is a stem loop structure that forms at the 5′ end of nascent viral RNA transcripts following transcription through the first 59 nucleotides [14]. Tat binds to TAR and recruits positive transcription elongation factor b (P-TEFb), a complex comprising cyclin T1 and cyclin-dependent kinase-9 (CDK9); the kinase subunit then hyperphosphorylates the CTD of the largest subunit of RNAP II, resulting in enhanced RNAP II processivity and transcription efficiency [15] (Figure 1a). In order to provide more insight into HIV pro-viral transcriptional regulation, Schulze-Gahmen and Hurley [22] determined the crystal structure of the super elongation complex in a complex with HIV Tat and TAR RNA. They observed that, in the crystal structure, the role of Tat is three-fold—namely, scaffolding and stabilization of the Tat-TAR recognition motif (TRM), making specific interactions through its zinc-coordinating loop and making electrostatic interactions through its arginine-rich motif (ARM). Interestingly, most recently, Hokello et al. [23] demonstrated that the cellular transcription factor, activator protein-1 (AP-1), synergizes with NF-κB to regulate HIV transcriptional elongation following T-cell receptor activation.

The HIV Tat provided the first example of viral gene expression regulation through the control of elongation by RNAP II. Although HIV Tat stimulates HIV transcriptional elongation primarily through specific interactions with TAR, and HIV-1 transcriptional regulation by Tat in most cell types requires intact TAR sequences [18], reports from several groups subsequently demonstrated that Tat regulation of HIV LTR-derived transcription in CNS-derived astrocytes and glial cells occurs in the absence of TAR, a mechanism of HIV transcriptional regulation referred to as TAR-independent transcription [24,25]. In this regard, numerous reports have subsequently demonstrated that the upstream transcription elements within the LTR were responsible for the Tat-mediated activation of HIV LTR transcription in the absence of the TAR element (Figure 1b). Specifically, the CNS-derived cells were found to exhibit kappa B-binding activity, which interacted with Tat to activate LTR transcription [24,26,27,28]. Indeed, the kappa B binding transcription factor from the CNS cells consisted of components that were indistinguishable from the prototypical NF-κB [26]. The TAR-independent Tat regulation of HIV transcriptional activity mediated by the kappa B-binding site provided implications with respect to the ability of Tat to alter cellular gene expressions and possibly contribute to a myriad of conditions associated with HIV infection, including the altered immune status, CNS toxicity, and tumor formation. Consistent with the above observations, other reports demonstrated that the HIV-1 Tat protein released from HIV-infected cells could be taken up by uninfected cells only to exert its effects on the responsive genes. For instance, Cupp et al. [29] and Sawaya et al. [30] demonstrated that HIV-1 Tat regulates the expression of transforming growth factor beta-1 (TGF-β1) in human astrocytic glial cells. TGF-β1 is a cytokine with potent immunosuppressive activity.

Gray et al. [31] also reported that the CNS-derived HIV-1 strains exhibit polymorphisms within the Sp1-binding sequence within the HIV LTR. These mutations resulted in decreased binding of the Sp1 transcription factor to the CNS-derived LTRs, thus reducing the transcriptional response of CNS-derived viruses. Accordingly, this observation suggests that the Sp1 transcription factor, which is critically important in HIV transcriptional regulation in many cell types, may not be required in HIV transcriptional regulation in cells derived from the CNS.

On the other hand, drugs of abuse, including cocaine, amphetamine, methamphetamine, heroin, and morphine, have been reported to upregulate HIV transcription, particularly in the CNS, and that illicit drug use correlates well with the high rate of HIV transmission among illicit drug users, as well as the rapid rate of acquired immunodeficiency syndrome (AIDS) progression [32,33,34,35]. Sahu et al. [36] demonstrated that cocaine, for instance, promotes both the initiation and elongation phases of HIV-1 transcription by activating NF-κB and mitogen and stress-activated kinase-1 (MSK-1). MSK-1 is responsible for the phosphorylation of the histone H3 and the p65 subunit of NF-κB, which subsequently enhances NF-κB interactions with p300, thus promoting the recruitment of P-TEFb. P-TEFb is a cellular cofactor for the viral Tat protein, which mediates HIV transcriptional elongation. In addition to the above elaborate mechanism of how drugs of abuse enhance HIV transcription, the observation that illicit drugs activate NF-κB is consistent with the fact that NF-κB mediates the TAR-independent Tat regulation of HIV transcription in cells derived from the CNS. Given these observations, it is clearly evident that HIV transcriptional regulation in the CNS is, in many ways, different from that of many cell types. This is especially so because different tissues are made up of specialized cells that are formed through cellular differentiation. As such, different types of cells contain different types of transcription factors, and this is the reason why there may be differential regulations of HIV transcription in the different anatomical sites represented in Table 1.

## 3. HIV Latency in the CNS

The latent HIV provirus, the hallmark of HIV latency, is integrated viral DNA that is transcriptionally silent and cannot be reached by the current combination antiretroviral therapy (cART), thus posing a great obstacle to the treatment and cure of HIV infection [37,38,39,40,41,42,43,44]. Attempts to cure HIV infection in patients undergoing cART require the effective targeting of all possible viral reservoirs in all anatomical sites. Other than the memory CD4+ T cells, several HIV reservoirs have been identified in different anatomical sites (Table 1) where HIV-1 continues to replicate despite cART. For instance, using the nonhuman primate model infected with RT-SHIV, a chimera of simian immunodeficiency virus containing the HIV-1 reverse transcriptase sequences, North et al. [45] demonstrated that HIV DNA and viral RNA were present in RT-SHIV-infected macaques that were treated with potent cART regimens consisting of efavirenz, emtricitabine, and tenofovir. Moreover, an additional analysis provided evidence for the presence of full-length viral RNA in tissues of the animals with the virus suppressed by cART. Interestingly, they showed that the highest levels of HIV DNA and viral RNA in cART-treated macaques were in lymphoid tissues—particularly the spleen, lymph nodes, and gastrointestinal tract—demonstrating that there is widespread persistence, as well as residual viral replication, in different anatomical sites, even during cART. In particular, the CNS is compartmentalized and serves as a specific site of HIV infection. Understanding of the cellular populations that harbor latent HIV infection in the CNS is critically important in attempts to find solutions to HIV latency.

HIV-1 infection of the CNS has peculiar characteristics in that CNS infection by HIV-1 can be significant and occurs very early following viral transmission. Secondly, local HIV replication in the CNS can be quite diverse and evolves over time. Thirdly, HIV-1 may persist in the CNS, even in the face of cART, due to insufficient penetration of the CNS by the current cART drugs but, also, due to the long-lived nature of the resident CNS cells. The physiological effects of HIV infection in the CNS are seen in patients with HIV-associated neurocognitive disorders (HAND) and HIV-associated dementia (HAD), which have been linked to increased neurological injury due to inflammation.

Early, following infection, HIV-1 can be found in the cerebrospinal fluid (CSF) at very low levels with very minimal viral burden in the CNS [46]. Therefore, this suggests that HIV most likely enters the CNS/CSF at low levels through the incomplete partitioning of the virus at the BBB or through background trafficking of the immune cells, including infected CD4+ T cells. Although the CNS is a site where HIV persists despite cART, persistence can also be maintained in the form of CSF escape, a scenario whereby HIV is detectable in the CSF but undetectable in the blood in some patients. For instance, Lustig et al. [47] reported a high prevalence of CSF escape of 28% in cART-treated HIV patients in South Africa. During another development, Peluso et al. [48] observed that the development of neurologic symptoms in HIV patients on cART with low or undetectable plasma viremia was an indication of CSF escape. The CSF, therefore, serves as the reservoir of the virus that replenishes the CNS or the blood.

Wallet et al. [49] observed that microglial cells, which are the resident macrophages in the CNS, are one of the major cellular reservoirs of HIV latency. Similarly, Ko et al. [50] demonstrated that it is the microglial cells that harbor HIV DNA in the brains of HIV-1-infected aviremic individuals on suppressive cART. Microglial cells are also the most abundant mononuclear macrophages found in the brain parenchyma. The median renewal rate of these cells is about 30% per year, and most of the cells in this population regenerate throughout a lifetime [51]. It is believed that microglial cells are infected by HIV-1 through the transmigration of infected monocytes, which occur very early in the course of HIV infection. Indeed, recent reports have identified a specific subset of HIV-infected monocytes that were HIV+, CD14+, and CD16+ that preferentially crossed the BBB [52]. Similarly, Leon-Rivera et al. [53] most recently characterized the mechanism of CNS viral reservoir establishment and replenishment using the peripheral blood mononuclear cells (PBMCs) of HIV patients on prolonged cART and established that monocytes from PBMCs with integrated HIV proviruses that were transcriptionally active selectively transmigrated across the human BBB model. In the CNS, replication-competent HIV-1 is reported to predominantly persist in resident macrophages, including microglia and perivascular monocytes/macrophages, whereby they disseminate the virus to other cell types. For a detailed review on the role of macrophages in the persistence of HIV pathogenesis, please refer to Kruize and Kootstra [54]. The infection and establishment of HIV latency in microglial cells seem to occur very early in the evolution of HIV infection [55]. The features of microglial cells, which allow for HIV persistence in the brain, include a resistance to cytopathic effects and the fact that they are nonlytic and resistant to apoptosis induced by HIV infection [56].

Using a cellular coculture system, the Karn group investigated the hypothesis that HAND resulted from the periodic emergence of HIV from a latency within microglial cells in response to neuronal damage or inflammatory signals. In this regard, when clonal human microglial cells (HC69) infected with HIV (hµglia/HIV) were cocultured for a short time with healthy neurons, HIV was silenced. However, the neuron-dependent induction of HIV latency in HC69 cells was demonstrated using induced pluripotent stem cell-derived GABAergic cortical and dopaminergic, but not motor, neurons, whereby damaged neurons induced HIV expression in latently infected microglial cells. Following 48–72 h of a coculture, low levels of HIV gene expression further damaged the neurons with a result that further enhanced HIV expression [57]. In a separate set of experiments, Alvarez-Carbonelli et al. [58] demonstrated that Toll-like receptor (TLR) signaling—in particular, TLR-3 activation—can efficiently reactivate HIV transcription in infected microglia but not in monocytes or T cells.

In another development, Llewellyn et al. [59] demonstrated that the corepressor to the RE1-silencing transcription factor (CoREST) were the key regulators of HIV latency in microglial cells in both rat and human microglial cells lines. Furthermore, they observed that monoamine oxide and the CoREST inhibitor phenelzine, which is capable of penetrating into the brain-stimulated HIV gene expression and production in human microglial cells recovered from brains of HIV-infected humanized mice models.

Astrocytes, which are the most abundant glial cells in the brain, are involved in brain plasticity and neuroprotection. The HIV infection of astrocytes in both cell cultures and brain tissues has been reported, and the current consensus is that the HIV infection of astrocytes leads to latent infection. Being the most abundant, long-lived cell types in the CNS, astrocytes are critically important in the maintenance of latent HIV reservoirs in the CNS. Li et al. [60] reported that the infection of astrocytes occurred through cell-to-cell contact with infected T cells. They further observed that this process occurred CXCR4-dependently but without the involvement of the CD4 molecule. In another study of HIV-infected brain tissues, HIV infection was detected in perivascular macrophages and astrocytes, although without p24 gag expression and with no other histopathological changes [61]. In this case, evidence of HIV infection in the brain was determined by nested polymerase chain reaction (PCR) for viral DNA, suggesting that these were latent infections. Although long-term studies of HIV-1 persistence in astrocytes are lacking, several studies have demonstrated that the HIV-1 infection of astrocytes results in latency that is capable of reactivation [62,63,64,65]. For instance, Tornatore et al. [63] demonstrated that, although HIV-1 could be induced in infected astrocytes, recovery of the active virus was only possible following a coculture of astrocytes with T cells. The foregoing section therefore presents sufficient evidence of HIV persistence in the CNS.

## 4. Neuro-HIV Drugs and Therapy in the CNS

Despite the fact that cART has reduced the incidence of HAD, a high prevalence of HAND still persists in the era of cART, ranging from asymptomatic neurocognitive impairment (ANI) to severe HAD [66]. Before the introduction of cART, HAD frequently developed in HIV-infected individuals with low CD4+ T-cell counts with concomitantly high HIV plasma viral loads. However, with the introduction of cART, the incidence of HAD, as well as that of AIDS, decreased [66,67]. However, mild-to-moderate forms of HAND still occur, despite long-term treatment with cART [67]. The prevalence of HAND among HIV-infected individuals is estimated to range between 15% and 55% [68]. It is, however, worth noting that, despite the persistence of HAND during cART, our understanding of the pathogenesis of the CNS damage associated with HAND remains poorly understood, although it is believed that the possible mechanisms of causation include sustained CNS inflammation and various host genetic factors, as well as neurotoxicity of the cART drugs [69,70]. Although the use of cART improves the longevity and immune functions in HIV patients, the brain remains a site for sustained HIV replication and, thus, an important target for antiretroviral drugs. Therefore, a clear understanding of how the current anti-HIV drugs interact at the level of the BBB and the blood–CSF barrier to reach the CNS are critically imperative for the maximization of viral suppression in the CNS and improvement of the overall clinical outcome, particularly where HAND is concerned. It would also enable the identification of inhibitors of drug uptake to the CNS and enable the development of novel anti-HIV drugs that can reach the CNS.

For the effective control of HIV replication, cART utilizes anti-HIV drugs from different classes, including protease inhibitors (PIs) (Table 2). Unfortunately, the vast majority of the current anti-HIV drugs in use have been demonstrated to exhibit poor CNS penetration and distribution, with PIs as the class of cART drugs having the worst pharmacokinetics and pharmacodynamics profiles in the CNS [71].

Contrary to this observation, however, Mdanda et al. [72] recently demonstrated that Zidovudine and Lamivudine, both of which belong to the class of nucleoside reverse transcriptase inhibitors (NRTIs), are both able to reach and localize in the brain regions that are targets of HIV neurodegeneration in the CNS using a Sprague–Dawley rat model. In a different set of experiments, Mdanda et al. [73] most recently also reported that alternative NRTIs, including Abacavir and Stavudine, were able to reach the brain regions that are strongly implicated in the progression of HAND, while Didanosine exhibited poor entry within the brain, suggesting that it is not recommended for the treatment of neuro-HIV infection. Thomas [71] also observed that non-nucleoside reverse transcriptase inhibitors (NNRTIs) such as Nevirapine and Efavirenz can readily enter the CSF. Despite these observations, there are currently no specific anti-HIV drugs for the treatment of HIV-associated CNS complications. However, sustained efforts are being made to develop novel anti-HIV drugs solely for this purpose. For instance, Amano et al. [74] recently reported the development of three newly designed CNS-targeting HIV-1 PIs—namely, GRL-083-13, GRL-084-13, and GRL-087-13, all of which are highly effective against wild-type HIV strains and clinical isolates but with minimal cytotoxicity.

The simian immunodeficiency virus (SIV)/pigtailed macaque model has enabled the characterization of the successive immunologic and viral parameters. This model offers many parallels to HIV infection, including the development of the characteristic CNS inflammation associated with HAND, but also correlates well with the high viral loads in the brain. Beck et al. [75] used this model to study HAND in the context of cART, whereby they tested a number of anti-HIV drugs, including both the old and newer cART regimens. Initially, SIV/pigtailed macaques were treated with ART1, which is composed of Tenofovir (PMPA), Saquinavir, Atazanavir, and the Merck integrase inhibitor L-8708/2, which successfully reduced the HIV viral loads in both the plasma and CSF to below the limit of detection. However, in the cases with HAND, sustained inflammation persisted in the brain despite cART efficacy, characterized by elevated levels of tumor necrosis factor alpha (TNF-α) and CCL2. Beck et al. [75] further compared the blood and CSF samples obtained prior to infection with those obtained at the terminal study time points, which represented the longest duration of a SIV replication control. In this regard, they observed strong parallels between their SIV/pigtailed macaques model and reports of HAND biomarkers identified in HIV-infected individuals receiving cART. For instance, in the CSF, there were elevated levels of both inflammation marker neopterin and the neuronal damage marker neurofilament light despite long-term cART. On the other hand, in plasma, soluble CD163, a monocyte/macrophage activation marker, and CCL2 were also elevated. Their findings indicated that SIV-infected pigtailed macaques receiving cART still developed persistent inflammation and neuronal damage, which closely correlated with the characteristics of HAND. Beck et al. [75] also tested a newer cART regiment, ART2, which consisted of PMPA, Darunavir, Ritonavir, and integrase inhibitor. This regimen also produced similar results to ART1. In order to refine the use of cART in SIV-infected macaques, Beck et al. [75] tested the efficacy of a third, newer regimen, ART3, comprising of a single once-a-day subcutaneous injection of PMPA, Emtricitabine (FTC), and Dolutegravir. They observed that this regimen rapidly suppressed the SIV/17E-Fr and SIV/DelaB670 strains of SIV in both the plasma and CSF of pigtailed macaques. Since plasma and CSF viral suppression was sustained over time, this model provided a long-term viral suppression model to study persistent CNS immune activation, as well as the neuronal damage that may underlie HAND.

Recently, the “Shock and Kill” approach has been proposed to eradicate viral reservoirs. The “Shock and Kill” approach involves the simultaneous treatment of HIV patients with latency reversing agents (LRAs) and cART drugs. Dental et al. [76] investigated the impact of different classes of LRAs on the integrity of the tight monolayers of the immortalized human cerebral microvascular endothelial cell line hCMEC/D3. Their results demonstrated that Prostatin and Bryostatin-1 significantly damaged the integrity of the endothelial monolayer in addition to inducing the secretion of some proinflammatory cytokines. Furthermore, they observed that these LRAs also enhanced the adhesion and transmigration of CD4+ and CD8+ T cells and monocytes in an in vitro human BBB model. The results of their study suggest that any attempts and efforts to purge latent HIV reservoirs from resting memory CD4+ T cells in the periphery should take into account the effects of such an approach on the integrity of the BBB, in addition to investigating its effects on HIV latency in other anatomical sites, such as the CNS.

Despite the problem of the limited or low penetration of most anti-HIV drugs in the CNS, the good news is that there are sustained research efforts to circumvent these obstacles in HIV therapy. Nanotechnology and nanomedicine possess the potential for therapeutics against neuro-HIV [77]. In this regard, Jayant et al. [78] developed a novel approach that involved the co-encapsulation of anti-HIV drug Tenofovir and LRA Vorinostat by using magnetically guided layer-by-layer (LBL) assembled nanocarriers for the treatment of neuro-AIDS. In this novel approach, ultrasmall iron oxide nanoparticles (10 +/− 3 nM) were synthesized and characterized, while the LBL technique allowed a sustained release and application of two layers of magnetic nanoparticles, which resulted in a 2.8-fold increase in drug loading and a 30-fold increase in sustained drug release. Using this same approach, Jayant et al. [79] also developed a novel nanoformulation to prevent the coeffects of drugs of abuse and HIV infection, considering the fact that drugs of abuse are one of the major risk factors for HIV infection and that a combination of illicit drug use and HIV infection can lead to significantly greater CNS damage and neurodegenerative disorders. Similarly, Rao et al. [80] demonstrated that the conjugation of nanoparticles to transcription trans-activator protein Tat peptides increased the transport of the encapsulated Ritonavir, a PI, across the BBB to the CNS. Asahchop et al. [81] reported that a reduced anti-HIV drug efficacy and concentration in HIV-infected microglia contributes to viral persistence. Therefore, the development and effective use of nanomedicine, particularly nanoART, could potentially help in the treatment of not only HAND but also purge out latently infected HIV proviruses from the CNS. 

## 5. Conclusions

There is clear evidence suggesting that there are distinct mechanisms of HIV transcriptional regulation between cells in the periphery, such as CD4+ T cells, and those of the CNS. For instance, whereas HIV transcriptional regulation in peripheral cells is majorly Tat-dependent through Tat interactions with the TAR RNA stem–loop structure, HIV transcriptional regulation in the CNS is TAR-independent, whereby the function of TAR is instead substituted by cellular transcription factor NF-κB, which is demonstrated to recruit Tat to the transcription machinery. The Tat-regulated but TAR-independent regulation of transcription provides implications on the expression of cellular genes. It is conceivable that this mechanism of HIV transcriptional regulation in the CNS may also be responsible for the observed increased in HIV replication in the CNS, even in the presence of prolonged cART, because basal levels of NF-κB may be sufficient to recruit Tat into the HIV transcriptional machinery. There is also mounting evidence to suggest that HIV latency is established in microglial cells and astrocytes in the CNS. Therefore, a clear understanding of the distinct mechanisms of HIV transcriptional regulation in different anatomical sites, including the CNS, is critically imperative in efforts geared towards purging latent HIV proviruses to achieve a functional cure for HIV infection.

Finally, while there is currently no standardized therapy for neuro-HIV and its associated complications in the CNS, a better characterization and understanding of the existing anti-HIV drugs that can cross the BBB, as well as the CSF barrier, with a subsequent increase in bioavailability in the CNS will enable the optimization of the available drugs for the treatment of neuro-HIV and its associated complications. However, the ultimate solution to the challenge of neuro-HIV therapy lies in the development of nanotechnology and its application in nanoART, whereby there is an urgent need to not only develop novel anti-HIV drugs that can cross the BBB but also optimize the use of nanocarriers to enable the better utilization of the currently available anti-HIV drugs in the therapy of neuro-HIV and its associated CNS complications.

## Figures and Tables

**Table 1:** The different anatomical sites that harbor latent HIV provirus reservoirs.

Anatomic Sites	Specific Sites
Brain	
Primary Lymphatic tissue/organ	Thymus and Bone marrow
Non-Lymphoid tissues	Liver, Kidney, Adipose, reproductive tract and other
Secondary lymphatic tissue/organs	Tonsils, Adenoids, spleen, Mucosa-associated lymphatic tissues and lymph nodes
Peripheral Blood	

**Table 2:** Showing the different classes of cART drugs currently in use for the treatment of HIV infection.

Target	Name of Drugs
Entry inhibitors	Maraviroc (Celsentri), Enfuvirtide
Nucleoside/nucleotide reverse transcriptase inhibitors (NRTIs)	Abacavir, Emtricitabine, Lamivudine, Tenofovir disoproxil, Tenofovir alafenamide, Zidovudine
Non-nucleoside reverse transcriptase inhibitors (NNRTIs)	Doravirine, Efavirenz, Etravirine, Nevirapine, Rilpivirine
Integrase inhibitors	Bictegravir, Dolutegravir, Elvitegravir, Raltegravir, Cabotegravir
Protease inhibitors (PIs)	Atazanavir, Darunavir, Lopinavir, Ritonavir (Norvir), Cobicistat (Tybost)
Attachment and post-attachment inhibitors	Fostemsavir (Rukobia), Ibalizumab (Trogarzo)

## Data Availability

Not Applicable.
